# Breast cancer therapy for BRCA1 carriers: moving towards platinum standard?

**DOI:** 10.1186/1897-4287-7-8

**Published:** 2009-04-20

**Authors:** Evgeny N Imyanitov

**Affiliations:** 1 N.N. Petrov Institute of Oncology, Pesochny-2, 197758, St Petersburg, Russia

## Abstract

Recently Byrski et al. reported the first-ever breast cancer (BC) study, which specifically selected BRCA1-carriers for the neoadjuvant treatment and used monotherapy by cisplatin instead of conventional schemes. Although the TNM staging of the recruited patients was apparently more favorable than in most of published neoadjuvant trials, the results of Byrski et al. clearly outperform any historical data. Indeed, 9 of 10 BRCA1-associated BC demonstrated complete pathological response to the cisplatin treatment, i.e. these women have good chances to be ultimately cured from the cancer disease. High sensitivity of BRCA1-related tumors to platinating agents has been discussed for years, but it took almost a decade to translate convincing laboratory findings into first clinical observations. With increasing stratification of tumor disease entities for molecular subtypes and rapidly growing armamentarium of cancer drugs, it is getting technically and ethically impossible to subject all promising treatment options to the large randomized prospective clinical trials. Therefore, alternative approaches for initial drugs evaluation are highly required, and one of the choices is to extract maximum benefit from already available collections of biological material and medical charts. For example, many thousands of BC patients around the world have already been subjected to second- or third-line therapy with platinum agents, but the association between BRCA status and response to the treatment has not been systematically evaluated in these women. While potential biases of retrospective studies are widely acknowledged, it is frequently ignored that the use of archival collections may provide preliminary answers for long-standing questions within days instead of years. However, even elegantly-designed, small-sized, hypothesis-generating retrospective studies may require multicenter efforts and somewhat cumbersome logistics, that may explain the surprising lack of historical data on the platinum-based treatment of BC in BRCA1 carriers.

## Introduction

On July 23, 2008, *Breast Cancer Research and Treatment *journal released an electronic publication ahead of print of the first-ever breast cancer (BC) study, which specifically selected BRCA1-carriers for the neoadjuvant treatment and used monotherapy by cisplatin instead of conventional schemes [1]. The results of the this exploratory trial are absolutely fascinating: 9 out of 10 patients experienced complete pathologic tumor response, so these women are expected to be relapse-free for a prolonged period of time or, hopefully, forever.

## Discussion

The limitation of the study of Byrski et al. [1] is an unusually favorable TNM staging of the treated population: 5 out of 10 females had tumor size below 2 cm, and 7 were lymph node negative. In comparison, retrospective analysis of Chappuis et al. [2], in which 4 out of 9 evaluable BRCA1 carriers demonstrated complete pathologic tumor response to anthracycline-based neoadjuvant therapy, included only 1 and 3 patients with T1 and N0 status, respectively. However, even upon some adjustment for the disease stage and moderate study size, the results of Byrski et al. [1] clearly outperform all known BC neoadjuvant trials.

Evidence for increased sensitivity of BRCA1-associated tumors to some chemotherapeutic agents started to accumulate nearly a decade ago, and the use of platinating compounds specifically for hereditary breast or ovarian cancers is being discussed in the literature for several years [see [3-9] and references therein]. Why it took so long to translate fairly convincing laboratory findings into clinical observations? First of all, relatively high efficacy of traditional drug combinations makes it difficult to justify a BC trial for a novel agent, unless heavily pretreated patients with advanced metastatic disease are involved. However, these women are less likely to demonstrate evident response even to a highly specific therapeutic intervention, due to acquired multidrug tumor resistance and general exhaustion of the body resources. In addition, selection of BRCA1 carriers for the trial possesses a problem because of rarity of BRCA1-associated BC (less than 5% of unselected BC patients) and high cost of BRCA1 testing.

These difficulties are reflected by ongoing randomized trial on BRCA1 and BRCA2 carriers, whose breast cancer disease progressed after adjuvant or palliative anthracycline-based therapy . The standard option for anthracycline-resistant BC is the use of taxanes. In the above trial of BRCA-associated BC, patients are randomized to receive either docetaxel (standard arm) or carboplatin (experimental arm). The study was launched in April, 2006  and is expected to complete the recruitment by October, 2009. The planned study size is 148 subjects; by the year 2008, 15 patients have been successfully recruited .

Byrski et al. [1] have chosen more decisive approach, benefiting from some favorable circumstances in Poland, e.g. well-established infrastructure for hereditary cancer diagnosis, large number of oncological patients undergoing routine DNA testing, and high impact of BRCA1 founder mutations in BC morbidity. Based on sound preclinical evidence for increased sensitivity of BRCA1-deficient breast cancer cells to platinating agents, this Polish-Canadian research team has taken a risk of recruiting potentially operable and yet chemonaive breast cancer patients for the study. Furthermore, while most of neoadjuvant schemes for BC treatment are based on combinations of several drugs, Byrski et al [1] left no room for ambiguous interpretation by deciding to use cisplatin as a monotherapy. Although long-term outcomes for these patients, particularly the response to the treatments in case of tumor relapse, remain to be seen, the study of Byrski et al [1] has to be considered as the first long-awaited clinical argument for the preferential use of platinating agents in BRCA1 carriers.

As for all human studies, the designs of the above 2 trials may be a subject of debate. One would argue, that the described randomized trial for carboplatin *versus *docetaxel is designed with perfect respect of the current treatment standards and is expected to provide highly conclusive data sets, but it is overly conservative and therefore it will take too long for the final results of this trial to become available. In contrast, Byrski et al. [1] offered an experimental treatment to those patients, who had a relatively good chances to be cured by already existing approaches; perhaps, the design of this neoadjuvant study could become a subject of harsh criticism if cisplatin failed to induce spectacular tumor responses in BRCA1 carriers.

With increasing stratification of tumor disease entities for molecular subtypes and rapidly growing armamentarium of cancer drugs, it is naïve to expect that all promising treatment options will be subjected to prospective randomized trials. Furthermore, the pipeline for novel smart antitumor molecules appears to work faster than the one for clinical trials. For example, the results of the randomized trial on the use of platinum compounds against BRCA-associated BC are unlikely to be obtained before the end of this decade; by that time, the data on PARP inhibitors will probably become available as well [9], and the latter drugs could have better chances to enter clinical settings because of more favorable safety profile [10]. In other words, platinum compounds may become outdated just after passing the test.

Therefore, alternative approaches for initial drugs evaluation are highly required, and one of the choices is to use retrospective biological material and medical charts [11]. For example, many thousands of BC patients around the world have already been subjected to second- or third-line therapy with platinum agents, but BRCA status has not been systematically evaluated in these women. If we hypothesize the advantage of platinum-based treatment for BRCA carriers, the most straightforward approach would be to collect archival material from responders, and to examine if the frequency of BRCA mutations is elevated in this rare category of patients. Interestingly, similar strategy was recently applied by Wysocki et al. [12], who limited BRCA1 analysis by BC patients with resistance to neoadjuvant docetaxel, and confirmed earlier clinical observations on the poor response of BRCA1-associated BC to taxanes [13]. Instead of selection of highly demonstrative categories of patients, one may also rely on communities with founder effect, where the analysis of BRCA status is cheap and high number of BC patients can be DNA-tested [14].

## Conclusion

While potential biases of retrospective studies are widely acknowledged, it is frequently ignored that the use of archival collections may provide preliminary answers for long-standing questions within days instead of years. However, even elegantly-designed, small-sized, hypothesis-generating retrospective studies may require multicenter efforts and somewhat cumbersome logistics, that may explain the surprising lack of historical data on the platinum-based treatment of BC in BRCA1 carriers.

## Abbreviations

BC: breast cancer.

## Competing interests

The author declares that he has no competing interests.

## Authors' contributions

ENI is the sole author of this manuscript.
